# Role of Extracellular Vesicles in Compromising Cellular Resilience to Environmental Stressors

**DOI:** 10.1155/2021/9912281

**Published:** 2021-07-20

**Authors:** Mona G. Alharbi, Seok Hee Lee, Aaser M. Abdelazim, Islam M. Saadeldin, Mosleh M. Abomughaid

**Affiliations:** ^1^Department of Biochemistry, College of Sciences, King Saud University, Riyadh, Saudi Arabia; ^2^Center for Reproductive Sciences, Department of Obstetrics and Gynecology, University of California San Francisco, San Francisco, CA 94143, USA; ^3^Department of Basic Medical Sciences, College of Applied Medical Sciences, University of Bisha, Bisha 61922, Saudi Arabia; ^4^Department of Physiology, Faculty of Veterinary Medicine, Zagazig University, Zagazig 44519, Egypt; ^5^Department of Medical Laboratory Sciences, College of Applied Medical Sciences, University of Bisha, Bisha 61922, Saudi Arabia

## Abstract

Extracellular vesicles (EVs), like exosomes, are nanosized membrane-enveloped vesicles containing different bioactive cargo, such as proteins, lipids, mRNA, miRNA, and other small regulatory RNAs. Cell-derived EVs, including EVs originating from stem cells, may capture components from damaged cells or cells impacted by therapeutic treatments. Interestingly, EVs derived from stem cells can be preconditioned to produce and secrete EVs with different therapeutic properties, particularly with respect to heat-shock proteins and other molecular cargo contents. This behavior is consistent with stem cells that also respond differently to various microenvironments. Heat-shock proteins play roles in cellular protection and mediate cellular resistance to radiotherapy, chemotherapy, and heat shock. This review highlights the possible roles EVs play in mediating cellular plasticity and survival when exposed to different physical and chemical stressors, with a special focus on the respiratory distress due to the air pollution.

## 1. Introduction

The bystander effect (BE) is a phenomenon where cells naïve to stress exhibit stress effects following exposure to signals from stressed cells [1]. This form of cellular communication manifests as an autoimmune response and amplifies possible side effects of a treatment or immune responses to a foreign antigen [2]. Remarkable progress has been made in the past 20 years towards understanding the roles of heat [1, 3, 4], inflammation [5, 6], radiation [7], chemotherapy [8, 9], and hormonal- and immunotherapies [10–12] in the BE. In particular, communication between different cells in the lung, such as bronchial epithelial cells, alveolar cells, endothelial cells, stem cells, and the immune cells, enables the respiratory system to maintain proper lung function [13, 14]. Unfortunately, smoking and continuous exposure to air pollutants, such as allergens and microbes, are the most harmful factors contributing to respiratory diseases [15–17]. Recent studies have revealed the pivotal role played by the mesenchymal stem cells (MSCs) plays in fighting tissue injuries by transferring information from the environment to stem and progenitor cell populations [18].

Increasing evidence suggests increases in temperature or environmental, physiological, or clinical stress conditions induce expression of a family of stress proteins called heat-shock proteins (HSPs). Members of the HSP superfamily play essential roles in protein folding, translocation, and degradation and were initially called “molecular chaperones” when first discovered in 1978 [19]. Heat-shock proteins are classified into various families based on molecular weight, like HSP60, HSP70, HSP90, HSP110, and small HSPs. Early studies initially revealed that HSPs were generated in response to heat-shock stress. Since then, studies have shown that HSPs have essential roles in assisting protein folding and refolding in cells and play roles in preventing apoptosis during radiotherapy, chemotherapy, and immunotherapy [20]. Specifically, studies have indicated that HSP expression is often increased in tumor cells and associated with tumor progression, including increased migration and metastasis [21].

Intracellular expression of HSPs in the pathology of many diseases has been studied for decades [22]. Literature also indicates that the presence of HSPs in the extracellular environment is an indicator of pathological conditions, such as cancers [23]. Other studies have also revealed that HSPs can be secreted in free form by cells undergoing necrosis, such as when mechanical disruption damages membrane integrity and releases HSPs [24], or secreted in membrane-bound extracellular vesicles (EVs), such as exosomes [25–28]. According to a recent statement from the International Society for Extracellular Vesicles (ISEV), EVs are lipid bilayer vesicles released by cells into the surrounding environment [29]. These vesicles can be categorized into three main groups: apoptotic bodies released during apoptosis, microvesicles formed by plasma membrane budding, and nanosized EVs called exosomes that are secreted by endocytosis [30, 31]. The generic term EVs is used in recent literature to refer to all secreted membrane vesicles; however, as shown in Table 1, EVs are highly heterogeneous in size, contents, and origin of secretion. The nature, contents, and abundance of the extracellular vesicle are influenced by the physiological or pathological state of the cell of origin [32]. Any external or internal cell stimuli can modulate molecular mechanisms and EV targeting by impacting EV production, cargo, and release. Extracellular vesicles can shuttle mRNA, microRNA (miRNA), proteins, and lipids to target cells, change gene expression, and regulate target cell behavior [29, 31, 33–37]. An *in vitro* study showed that EVs released by irradiated cells can induce the BE in naïve cells, possibly by transferring their cargo [1].

**Figure 1:**
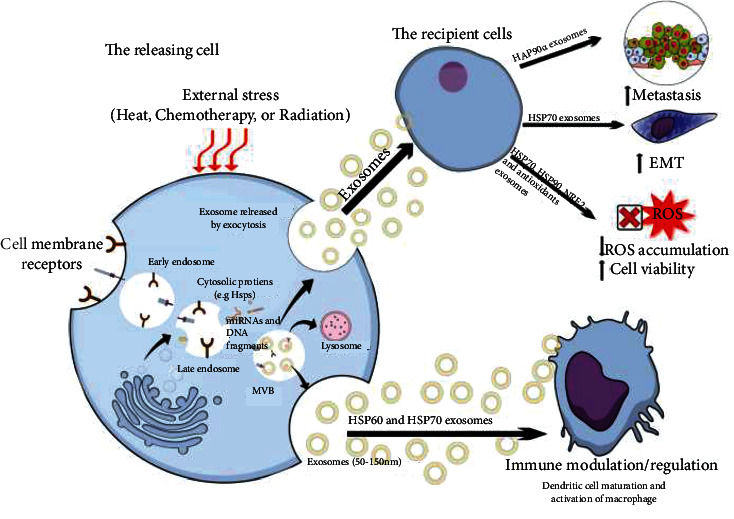
The role of exosomes and EVs in communicating the cellular bioactive signals between the releasing and recipient cells. Despite the bystander effects, stressed cells release exosomes enriched with heat-shock proteins (HSPs) are up taken by the target cells to modify their functions, such as metastasis, oxidative stress, and immune modulation. ROS: reactive oxygen species; EMT: epithelial-mesenchymal transition; MVB: multivesicular body. Details about the signaling molecules are mentioned in Table 1.

Unlike microvesicles and apoptotic bodies that pinch off from the plasma membrane, exosome secretion requires biogenesis of multivesicular bodies (MVBs) and sorting of contents that delays exosome release. This delay may facilitate extra regulatory checkpoints for exosome secretion compared to microvesicles [32]. The term exosome has emerged in literature over the last two decades to describe a new cell factor that plays a crucial role in intercellular communication in normal and pathological states. Exosomes are approximately 50-150 nm [38] nanovesicles released from originating cells into the extracellular environment via the endocytic pathway. Biogenesis of exosomes begins with inward budding of the cell membrane that captures some cellular membrane receptors to form an early endosome. Invagination of the endosome integrates various cytosolic proteins and nucleic acids, including mRNA, miRNA, and other noncoding RNAs, to form MVBs that can fuse with the cytoplasmic membrane and release exosomes or fuse with the lysosome for degradation [33]. The regulatory mechanisms that confer degradation in lysosomes or secretion of MVBs as exosomes are largely unknown. However, there is a balance that determines cell function and communication [32]. Lysosome inhibition increases exosome production indicating that exosome release may compensate for impaired lysosomal functions or help dispose of misfolded proteins generated under pathological or physiological conditions [39]. Released exosomes are small copies of originating cells [40] enriched with biomolecules, like proteins, specific to the originating cells (Figure 1).

Proteins that are enriched within exosomes or on the exosomal membrane include tetraspanins (e.g., CD9, CD63, and CD81), the Rab family of proteins that control exosome release (e.g., Rab27a and Rab27b), tumor oncogenes or suppressors, proteins associated with MVB biogenesis (e.g., Alix and TSG101), and heat-shock proteins (e.g., Hsc70 and HSP90) [41]. Exosomes are found in all biological fluids and can target either nearby or distant cells influencing recipient cell metabolism; subsequently, an association has been established between exosomes and the BE. For example, EVs transport bioactive molecules, including nucleic acids, proteins, and lipids influencing intercellular communication and inducing cancer progression and immune remodeling [42]. Moreover, EVs mediate the BE for lung diseases and lung regeneration [18]. Therefore, this review will examine evidence substantiating the roles of HSPs and exosomes in promoting the BE and remodeling target cells. This review will also discuss different EV profiles and contents and how these variable factors induce the BE and modulate metabolic responses in the recipient cells.

## 2. Heat-Shock Proteins

Heat-shock proteins (HSPs), also referred to as bystander antigens or chaperones, are a large family of chaperone proteins categorized by molecular weight into six major families [43]. In nonstressed cells, HSPs are expressed as intracellular proteins in low levels [44]; however, during stress conditions, HSPs are produced when stress induces heat-shock factor that binds to heat-shock elements in HSP promoters in DNA, leading to the synthesis of HSPs [43]. Studies have shown that HSP families are located in different sites in the cell and exhibit different functions. For example, HSP10, HSP60, and HSP75 are localized in mitochondria, while other HSPs are present in the cytoplasm, cytosol (HSP70 and HSP90), endoplasmic reticulum, and nucleus (HSP90) under physiological conditions [45]. HSP70 and HSP90 have cytoprotective functions inside cells [46, 47], whereas HSP60 together with its cochaperone HSP10 is essential for mitochondrial protein folding [48].

Over the past several years, HSPs have been shown to play an essential role in cell survival under conditions of stress. In addition to intracellular functions, HSPs have extracellular functions when secreted in free form from necrotic cells [49], encapsulated inside exosomes released into the extracellular environment [25, 50, 51], or secreted into the circulation during inflammation, like HSP60 [52]. The most studied HSP families are HSP90, HSP70, HSP60, and HSP100. In particular, HSP70 is the most characterized family because of its highly conserved proteins from prokaryotes to eukaryotes [53]. The HSP70 family has also been categorized into stress-induced or nonstress-induced members due to HSP70s being expressed either intrinsically or inductively [54, 55]. In the development of cancer biomarkers, HSP90 is a promising candidate for cancer diagnosis, prognosis, and prediction of responses to treatment [56]. Other studies have shown that HSP70, HSP60, and small HSPs could be used to treat neurodegenerative diseases, ischemia, and autoimmunity [57]. However, a critical question remains regarding what roles HSPs play in intercellular communication.

## 3. Paradoxical Role of Heat-Shock Proteins

In nonstressed cells, HSPs are expressed either in very low amounts or not at all [58]; however, under conditions of stress, HSP expression is induced. Following cellular induction, HSPs function in paradoxical roles that depend on the site of release. Precise HSP induction mechanisms remain unknown; however, the effects of HSPs can be divided into intracellular release (cytoprotective/antiapoptotic functions) or extracellular release (immunomodulation) effects [44, 59]. Intracellular HSP release has been shown to activate multiple steps in the apoptotic pathway to prevent cell death. For example, intracellular release of HSP70 inhibits apoptosis signals by interacting with procaspase 3 and procaspase 7 to block apoptosis. Another study reported that HSP70 suppresses the apoptotic pathway by inhibiting cytochrome c release from mitochondria [60, 61]. As a result, HSP70 plays a tumorigenic role because of its antiapoptotic properties [44].

In a recent study, heat stimulation was found to alter expression levels of mitochondrial HSP60 and activity of electron transport chain complexes [62]. Past studies suggested that the function of HSPs as chaperones is to maintain protein homeostasis [63], and when released into the extracellular environment, HSPs initiate an immunogenic effect through the chaperoning of antigenic peptides [64]. In particular, HSP70 can trigger an active response from intact cells of the immune system following stress as HSP70 partially diffuses into the plasma membrane and promotes ion channel formation before being released within EVs [65, 66]. The presence of HSPs in the extracellular environment is thought to be an indicator of cellular injury implicating HSPs in inflammation and immunity [65].

The main roles of circulating small HSPs in health and disease remain unclear even though high serum levels of HSP27 have been reported in several diseases, including hepatocellular tumors [67], thyroid papillary carcinoma [68], and pancreatic carcinoma [69]. A recent study also found that HSP60 is highly expressed in Graves' autoimmune disease and induces inflammation along with disease pathogenesis [70]. Accumulation of HSPs in exosomes is one mechanism through which HSPs appear in circulation, as evidenced by the presence of HSP27 in exosomes during heat stress [25]. Several studies have also indicated that extracellular secretion of HSPs might play a role in stress signaling, angiogenesis, cell migration, and immune modulation/regulation [56, 71, 72].

## 4. Bystander Effect and Exosomes

A number of factors, such as heterogeneous nuclear ribonucleoprotein A2B (or hnRNPA2B) [73], Y-box protein [74], and AGO2 (Argonaute2) [75], have been implicated in the sorting of exosomal cargo. Posttranslation modifications, such as ubiquitin, the addition of peptides, and sumoylation, have also been suggested to control sorting during exosome biogenesis [76]. The packaging of bioactive molecules within exosomes is a complex process with the resultant cargo reflective of the physiological state of the originating cell. Hence, any damage that alters the physical and chemical properties of a cell will be transferred into exosomes during synthesis [77]. Investigating the protein composition of exosomes might reveal the cell origin of exosomes and provide insights into the cell state (e.g., if the cell was under stress). Research interest in exosomal contents has grown in recent years due to the role exosomes play in intercellular communication.

Recent advances in the proteomic analysis of exosomes have helped identify protein components of exosomes, determine cell origins, and ascertain the state of the originating cell. For example, proteomic assessment of exosomes from cancerous cells has revealed the presence of proteins associated with tumor progression [78], such as metastasis [79, 80], angiogenesis [81], and signal transduction [82]. Stress conditions also impact mRNA and protein composition of vesicles released by stressed cells, as evidenced by the functional importance of such changes in the context of cell responses to stress [83, 84]. Previously, HSP70 was detected within the plasma membranes of tumor cells before being released into the extracellular environment in three forms: free soluble, complexed with peptides, and packaged into exosomes [85]. A recent study demonstrated that tumor-derived exosomes containing HSP70 exit cells through the plasma membrane [86]. Moreover, tumor-derived exosomes exclusively possess HSP70 compared to normal cell-derived exosomes [87] indicating that HSP70 might elicit innate or adaptive immune responses in favor of cancer cells. A study also showed that when primary amnion epithelial cells were grown under oxidative stress conditions, exosomes became enriched with HSP70 and P-p38 MAPK [88]. Similarly, stressed retinal pigment epithelium cells were found to produce exosomes containing seventy-two proteins associated with apoptosis and cell survival [89].

Exosomes play a crucial role in intercellular communication with environmental stress reported to influence the profile of released exosomes [90]. For example, exosomes isolated from conditioned media derived from heat-shocked cells were found to more strongly induce DNA damage and apoptosis in naïve cells compared to control exosomes. The same study showed that exosomes enriched with HSPs enhanced the invasive capacity of naïve cells [1]. The author of this study suggested that exosomes mediate intercommunication to alert neighboring cells and promote adaptive and more robust responses to heat stress in targeted cells [1]. Similarly, a study reported that the number of secreted exosomes increased, and the composition of exosomes changed, when B-cells were exposed to heat stress, compared with control cells. In the same study, exosomes were enriched with specific HSPs, like HSP27, HSC70, HSP70, and HSP90, but HSP60 was absent [25].

A recent cancer study also reported that HSP70, HSP90, and HSP60 were secreted by cancerous cells via exocytosis and might play a key role in inhibiting host immune responses against cancer cells [87, 91, 92]. Furthermore, extracellular secretion of HSP70 induces immunomodulatory effects in targeted cells and plays a key role in the immune response to cancer cells [12, 46]. Microvesicles loaded with HSP70 are also capable of activating macrophages [65] or natural killer cells [51] and play a key role in the regulation of epithelial mesenchymal transition [93]. Another HSP, HSP90*α*, is abundant in exosomes derived from invasive cancer cells and enhances cancer cell migration in recipient cells [94]. For instance, cancer cell-derived EVs were found to induce macrophage polarity in breast cancer [95], ovarian cancer [96], and pancreatic cancer [97]. A recent proteomic assessment of EVs in cancer cells revealed enrichment of a large amount of EVs with HSP90 from highly metastatic oral cancer cells [26], while HSP60 localized outside of cells was found to mediate interactions between immune cells and other body tissues [46]. In addition, recent experimental data indicate that HSP60 can be localized in extramitochondrial sites. In particular, HSP60 has been detected in the cytosol, inside intracellular vesicles, and on the surface of normal and tumor cells [46].

Exosomes isolated from X-ray-irradiated cell-conditioned medium have been shown to cause DNA damage in fibroblast cells. An *in vivo* study involving mice found that circulating exosomes extracted from irradiated mice contained mtDNA that induced DNA damage in nontreated mice [98]. Thus, it is possible that HSPs within exosomes might play a role in immunity. For example, ascitic fluid exosomes from cancer patients were found to be enriched with HSPs and act as effective antigens for dendritic cell cross-presentation to CD8^+^ T cells [99]. Similarly, exosomes derived from tumor cell lines were reported to be loaded with a large quantity of MHC-class I and Hsc70 and to function as immunologically effective vehicles for delivering antigens to dendritic cells [100]. These studies illustrate how any amount of cell damage can induce production of EVs and influence the physical and chemical properties of the resultant vesicles. Importantly, these studies demonstrate a link between stress and exosomal cargo. However, it is necessary to first understand changes in exosome composition following cell exposure to stress, before understanding the precise role exosomes play in regulating the BE.

## 5. Extracellular Vesicles Mediate Cell Resilience and Tolerance

### 5.1. Chemotherapy Resistance

Drug resistance remains a significant clinical problem hindering the success of any cancer treatment. The underlying mechanisms of drug resistance remain unknown, and currently, no reliable method exists that can predict tumor response to treatment without an invasive biopsy [101]. However, studies suggest that the changing phenotype of cancer cells might be detectable during cell-to-cell transfer of EVs, such as exosomes, in a process termed cross-chemoresistance [102]. This issue is now being tackled in cancer treatment studies by investigating the possibility of collecting EVs from biological samples in a minimally invasive method for monitoring cancer cell gene expression changes and for predicting clinical outcomes. Specifically, exosomal contents are being explored as potential biomarkers for patient responses to a particular treatment. Exosomes derived from cancer cells, unlike exosomes from normal cells, carry HSP70 on their membranes [103]. Exosomal HSP70 derived from serum also reflects HSP70 expression in tumor biopsies, indicating exosomal HSP70 enrichment associates with disease progression. Interestingly, HSP70-exosome levels are negatively correlated with poor patient treatment outcomes. This finding suggests that exosomal HSP70 might be useful for predicting tumor responses and clinical outcomes [103].

A study conducted on exosomes derived from conditioned media from ovarian cancer cell lines with different aggressive phenotypes revealed increased expression of specific miRNAs (miR-21-3p, miR-21-5p, and miR-891-5p) that correlated with aggressive phenotype [104]. In addition, the levels of these miRNAs increased in exosomes derived from carboplatin resistant cells. Moreover, circulating exosomes from patients with ovarian cancer showed a strong association between cancer recurrence and expression of miR-891-5p. Subsequent *in vitro* studies indicated that miRNA891-5p induced resistance by activating DNA repair mechanisms [104]. Similarly, preexposure of platinum-sensitive cancer cells to exosomes derived from resistant ovarian cancer cells reduced the platinum sensitivity profile of the recipient cancer cells and induced the epithelial-mesenchymal transition [102].

In a different study, the number of EVs significantly increased in breast cancer cells exposed to paclitaxel and doxorubicin and also became enriched with the cell signaling protein annexin 6 (ANXA6) that enhanced premetastatic capacity *in vitro*. The same study also showed enrichment of ANXA6 in circulating EVs obtained from patients undergoing neoadjuvant chemotherapy [105]. Exosomes derived from pancreatic cancer cells (PC) treated with gemcitabine (Gem-exosomes) also induced acquired chemoresistance when coincubated with naïve PC cells. Proteomic and miRNA analyses showed that Gem-exosomes were enriched with reactive oxygen species- (ROS-) detoxifying genes, such as superoxide dismutase 2 (SOD2), catalase (CAT), and miR-155, when compared with control exosomes. Coincubation of Gem-exosomes with PC cells also reduced cell sensitivity to gemcitabine. However, PC cells transfected with a miR-155 inhibitor exhibited sensitivity to gemcitabine, even after coincubation with Gem-exosomes [106].

Several studies have indicated that changes in cell phenotypes might be reflected in EV cargo, and that EVs can interact with naïve cells and induce resistant phenotypes. This ability to package bioactive molecules into EVs might be useful for detecting circulating biomarkers that reflect changes in microenvironmental conditions and for predicting responses to treatment. Interestingly, combining heat stress and chemotherapy with doxorubicin increased the number of doxorubicin-containing exosomes from breast tumor cells. Furthermore, the resultant exosomes enhanced *in vitro* and *in vivo* antitumor effect [107]. That is, heat stressed and doxorubicin-containing exosomes inhibited MCF-7 tumor cell proliferation and triggered MCF-7 cell apoptosis in a pathway associated with increased expression of caspases 3 and 8 and inhibited the growth of implanted breast tumors in mice [107, 108].

### 5.2. Radiotherapy Resistance

Radiotherapy induces post or precancer debulking in conjunction with chemotherapy or immunotherapy. Studies have shown a strong effect of radiotherapy as a local cancer treatment through induction of cell apoptosis. Recent *in vitro* studies showed that the cells not directly targeted by ionizing radiation accumulated genetic alterations mediated by soluble factors released by irradiated cells [109]. This phenomenon is known as the radio-induced bystander effect that induces genetic instability, apoptosis, and senescence to stress, including radiation exposure [110]. Recently, it has been suggested that these BEs are closely related with release of EVs derived from irradiated cells [111]. Irradiated cancer cells undergoing apoptosis release EVs or exosomes capable of inducing BEs in nonirradiated neighboring cells. These exosomes promote genetic instability and provoke treatment resistance in recipient cells [112]. For example, treatment with the conditioned medium from murine gamma-irradiated lymphocytes increased cell survival, reduced cell apoptosis, and protected lymphocytes from irradiation-induced lethal responses [113]. This adaptive response was mediated through the transfer of soluble factors into the conditioned medium, such as those associated with the phosphatidylinositol 3-kinase (PI3K)/AKT signal pathway [113]. Moreover, Sarin et al. [114] demonstrated how EVs derived from irradiated neuroblastoma cells activate survival pathways, enhance cellular proliferation, and accumulate recipient cells in the G2/M phase of the cell cycle (Table 2).

### 5.3. Heat-Shock Resistance

Circulating HSPs may be involved in tumor growth. For example, cancer cells under stress, such as hypoxia, acidosis, and nutrient deficiency, have been found to induce expression of HSP90 [115]. This HSP plays a role in stimulating tumor progression and metastasis in several types of cancers, including pancreatic cancer, breast cancer, and leukemia, and has been associated with poor clinical outcomes [115]. A lack of HSP90*α* on exosomal membranes may impair the capacity of exosomes to act as important intercellular mediators between tumor cells and stromal cells [116]. In an *in vitro* study, HSP90*α* expression increased in prostate cancer cells compared to normal prostate epithelial cells [117]. In addition, when prostate cancer cells were exposed to 45°C (i.e., heat stress), more HSP90*α* was released into the conditioned medium as free HSP90*α* and encapsulated within EVs [117]. This *in vitro* study also showed that the size of EVs increased in response to heat shock from 50 to 200 nm, without heat shock, up to 200–500 nm 0.5, 1.5, and 3 hours after heat shock. Furthermore, when expression of HSP90*α* was inhibited in prostate cancer cells, E-cadherin expression increased suggesting that HSP90*α* is crucial in cancer epithelial-mesenchymal transitions (EMTs). The size of EV particles also decreased following inhibition of HSP90*α* expression [117]. In the *in vivo* study, a triple knockdown of chaperones CDC37, HSP90*α*, and HSP90*β* inhibited tumorigenesis of pancreatic cancer cells [117]. Another study showed that levels of HSP70 exosomes are higher in plasma from breast and lung cancer patients in comparison to control groups [118]. Similarly, HSP70 exosomes were found to increase in metastatic lung cancer from nonmetastatic patients [118]. This study also showed that HSP70-containing exosomes from the metastatic group exhibited high levels of stress proteins [118]. Exosomal membrane levels of HSP70 were also elevated in stage IV melanoma patients [93]. Several studies suggest that levels of HSP-positive EVs in plasma could be used as a predictive biomarker for early disease or as prognostic biomarker for disease progression (Table 3).

The HSP proteins HSP70, HSP90, HSP60, and HSP27 are reported to be associated with EV synthesis in response to heat shock [119]. Several genes involved in the stress response pathway are closely related with heat-shock factors in response to heat-shock stimuli [120]. Heat-shocked cells (MCF-7and K562 cells) that received EVs from heat-shocked cells showed significantly lower levels of DNA damage and apoptosis compared to heat-shocked cells pretreated with control-cell conditioned medium [1]. Similarly, heat stress-related EVs induced adaptive responses in cultured bovine granulosa cells exposed to heat shock. This observation was evidenced by improved granulosa cell viability and decreased ROS accumulation and was mediated through transfer of HSP90, HSP70, NRF2, antioxidants, and several other microRNAs [121, 122].

In addition to using HSPs for disease diagnosis or prognosis, several studies have explored the therapeutic potential of HSPs by investigating correlations between HSPs and miRNAs. In cervical cancer cell lines, miR-361 was found to regulate HSP90*α* expression and suppress EMTs [123]. Triptolide, an inhibitor of HSP70, induces miR-142-3p expression that inhibits proliferation of pancreatic cancer cells [124]. Similarly, decreased miR-29a expression was associated with the apoptotic level of breast cancer cells through upregulation of HSP60 and downregulation of HSP90, HSP70, HSP27, and HSP40 [125]. Conclusively, many studies demonstrate a key role for miRNAs in HSP expression. Therefore, understanding the roles of HSPs and miRNAs in specific diseases may reveal new treatment options.

### 5.4. Immune Response and Cancer Vaccine via Heat-Shock Proteins in Exosomes

Studies present conflicting results regarding the release of HSP-containing exosomes from tumor cells. For example, exosomes were found to mediate HSP70 and play a pivotal role in cross-priming T lymphocytes [126]. Conversely, tumors cells (EL4 thymoma) released HSP72-containing exosomes and exerted immunosuppressive actions through interactions with myeloid-derived suppressor cells [87]. Additionally, dimethyl amiloride decreased exosome production by tumor cells and enhanced the antitumor efficacy of the chemotherapeutic drug cyclophosphamide in three different mouse tumor models through TLR2-dependent pathways [87]. Interestingly, exosomes derived from heat-treated malignant ascites of gastric cancer patients contained HSP70 and HSP60 that promoted dendritic cell (DC) maturation and induced a tumor-specific cytotoxic T lymphocyte (CTL) response [127]. Moreover, heat-shocked tumor exosomes that contained high levels of HSP70 exhibited antitumor effects and converted regulatory T cells into T helper type 17 cells via an IL-6-dependent process [128]. Furthermore, tumor hyperthermia potentiated immune system responses through release of exosomes containing chemokines (CCL2, CCL3, CCL4, CCL5, and CCL20), HSPs, and tumor antigens to the antigen-presenting cells and facilitated tumor cell attack and tumor cell surface modulation [129–132].

### 5.5. Oxidative Stressed Cells Protect Unstressed Cells

In a study involving exosomes released by mouse mast cells (MC/9 cells) exposed to hydrogen peroxide-induced oxidative stress, exosomes released from the stressed mast cells stimulated resistance to oxidative stress in naïve recipient cells. In the same study, exosomes contained shuttle mRNA and provided recipient cells with resistance against oxidative stress through enhanced cell viability [133]. In other studies, EVs from released from cells exposed to oxidative stress also reduced transepithelial resistance in naïve recipient cells. For instance, EVs derived from stressed retinal pigment epithelial cells mediated transfer of stress messages to recipient cells through surface ligands [134, 135]. Interestingly, EVs from stressed pigment cells can communicate stress messages to naïve cells and contribute to the dysfunction of retinal pigment epithelial cells in senescence and disease [134, 135]. Likewise, bovine granulosa cells treated with hydrogen peroxide released exosomes enriched with Nrf2 (mRNA and protein) and antioxidant molecules (CAT, PRDX1, and TXN1) and were capable of protecting naïve cells from oxidative stress [136].

### 5.6. Extracellular Vesicles Mediate and/or Antagonize Air Pollution Respiratory Effects and Respiratory Disorders

**Figure 2:**
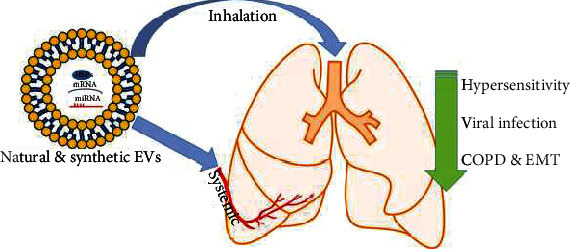
The potential used of natural and/or synthetic EVs in treating lung diseases. EVs contain proteins (such as HSPs), mRNA, and miRNA that play a pivotal role in reducing hypersensitivity, secreting mucin to antagonize viral infection and to reduce the epithelial-to-mesenchymal transition (EMT) that is associated with chronic obstructive pulmonary disease (COPD).

Extracellular vesicles derived from bronchial epithelial cells are major determinants of lung homeostasis that regulate the innate immune response inside lung tissue [137]. EVs contain many elements, like mucin (MUC-1, MUC-4, and MUC-16) and miRNAs (miR-34a/b/c, miR-449b/c, and miR-223), that play important roles in viral infections by modulating the qualitative and quantitative profiles of airway secretions, including MUC hypersecretion [138]. Importantly, EV miRNA contents can be used to measure pollutant exposure. For example, a study showed that the concentration of salivary EV microRNA correlates with the personal exposure to different doses of particulate matter and black carbon dose [139]. Interestingly, a study also found that EV output was reduced by cigarette smoke (CS) or exposure to cigarette smoke, suggesting that EVs do not play protective roles in lung inflammation [140]. Recent studies have also shown that EVs contribute to chronic obstructive pulmonary disease (COPD) by modulating the EMT process. Cigarette smoking also induces exosomal miR-21 that triggers differentiation of bronchial epithelial-myofibroblast differentiation through the von Hippel–Lindau protein/hypoxia-inducible factor 1*α* signaling pathway [141]. Importantly, downregulation of miR-21 appears to prevent airway remodeling [141]. Furthermore, high levels of exosomal miR-21 were found in sera of smokers and COPD patients and are inversely correlated with Forced Expiratory Volume in one second/Forced Vital Capacity (FEV1/FVC). These findings demonstrated an important role of exosomal miR-21 in the diagnosis and treatment of COPD. Recently, EVs derived from bronchial cells with less miR-21 alleviated M2 macrophage polarization and indirectly modulated the EMT process in the COPD [142]. A previous study also showed that miR-210 can be transferred to exosomes derived from bronchial fibroblasts and induce myofibroblast differentiation [143] (Figure 2).

Interestingly, EVs derived from MSCs prevented the accumulation of Ly6C^high^ monocytes and reduced the production of several cytokines, including TNF-*α*, IL-6, TGF-*β*, and IL-10 that mediate lung inflammation and fibrosis [18, 144]. The therapeutic capacity of EVs derived from MSCs can be enhanced by exposing MSCs to hypoxic and ischemic conditions—this provides evidence for the BE in MSCs and lung diseases [145].

Conversely, asthma is a chronic inflammatory disease induced by allergens and air pollutants that is characterized by airway hypersensitivity and reversible obstruction of airways. Extracellular vesicle miRNAs, like subsets of let-7 and miRNA-200 families, are altered in asthmatics [146, 147]. Level of EV miRNAs increases in the epithelium of airways exposed to allergens, particularly miR-223 and miR-142a that have been found to increase in mice treated with allergens [148]. Moreover, miR-142-3p, miR-629-3p, and miR-223-3p are elevated in the sputum of patients with severe asthma compared to healthy subjects [149]. A study involving inflamed tissue has also shown that infiltrating immune cells may alter the local extracellular environment via the release of EV miRNAs. In addition, several miRNAs are dysregulated in exosomes from the inflamed tissue of asthmatic patients [150]. Interestingly, EV miR-150 inhibits hypersensitivity responses in mice exposed to allergens and could be a promising therapeutics for allergic patients [151].

Extracellular vesicles may mediate other diseases with adverse respiratory symptoms, such as cardiovascular diseases, rheumatoid arthritis (RA), and possibly lung cancer (reviewed in [152]). Notably, particulate matter with aerodynamic diameters ≤10 *μ*m (PM10) and ≤2.5 *μ*m (PM2.5) exposure stimulated the release of EVs containing human endogenous retrovirus w (HERV-w) and human leukocyte antigen G (HLA-G) in RA patients [153].

## 6. Extracellular Vesicle Heat-Shock Protein and miRNA Cargo: A Therapeutic Target Worth Considering

Exosomes are among the most commonly known extracellular vesicles and represent snapshots of intracellular pathways. This may contribute to the dual action of these cell-derived exosomes. The development of therapeutics using exosomes is emerging and could be further potentiated when using stem cells to attain advantages of stemness together with benefits provided by HSPs. Recent studies showed that heat shock of Sca-1(+) stem cells induced exosome expression of HSF1 and HSP70 that converted ischemic cardiomyocytes toward a prosurvival phenotype through epigenetic repression of miR-34a [154]. Additionally, transplantation of heat-shocked Sca-1(+) stem cells and their exosomes significantly reduced apoptosis, attenuated fibrosis, and improved global heart functions in ischemic myocardium [154]. Furthermore, heat shock-derived exosomes can prime the response and therapeutics of stem cells. In comparison to controls, pretreatment of bone marrow mesenchymal stem cells (BMSCs) at 42°C for 1 hour showed the lowest apoptotic rate, decreased the number of autophagosomes, and reduced the expression of Beclin1 and the LC3BII/LC3BI ratio even after addition of cisplatin [155]. The cisplatin-induced apoptotic rates of granulosa cells were lower when cocultured with heat-shocked BMSCs due to high levels of HSP90 and HSP70 suggesting a therapeutic priming of exosomes from heat-shocked stem cells [155].

Similarly, mesenchymal stem cell- (MSC-) derived EVs possess an immunomodulatory power to treat asthma. Recently, MSC-derived exosomes have been found to upregulate the release of cytokines IL-10 and TGF-*β*1 from peripheral blood mononuclear cells, thereby stimulating the proliferation and immune-suppression capacity of T regulatory cells [156].

In general, methods used to isolate exosomes or any EVs are highly dependent on the aim of a study. However, the lack of a universal method for isolating pure populations of exosomes and the inconsistencies among methods for exosome enrichment in the literature are obstacles that have impeded progress in this field. More research is needed to determine the effects of different exosome isolation methods on the consistency of findings and reproducibility of data [157]. Differential centrifugation followed by ultracentrifugation is the most common method used for the enrichment of EVs; however, ultracentrifugation pellets contain EVs and other protein contaminants in solution (i.e., protein complexes and protein-miRNA complexes) [40].

To produce a pure population of vesicles, ultracentrifugation is combined with other methods, such as size exclusion chromatography or immunoprecipitation. Size exclusion chromatography involves separating EVs from the protein contaminants based on size; however, lipoprotein contamination cannot be resolved using size exclusion [40]. Immuno-affinity represents a promising method for producing a pure population of exosomes through the use of an exosomal protein-embedded membrane, such as CD63, to capture and collect exosomes [41]. High yields of exosomes will also help determine whether proteins are associated with exosomes or coprecipitate with the exosome population. Therefore, to ensure the success of future exosomal research, standardized methods for isolating pure populations of exosomes with minimal contamination by proteins and lipoproteins must be developed. This is an important area in exosome research that should be addressed before exosomes can be utilized as diagnostic biomarkers or biological targets.

Recent approaches are interested in manufacturing synthetic or semisynthetic EVs for targeted drug delivery. The drugs include proteins and nucleic acids (RNA, miRNA, and siRNA) can be loaded into the EVs through different methods such as electroporation, active loading with saponins, sonication, freezing/thawing, or through extrusion [158, 159]. Importantly, the trend in using EVs for lung treatment is still in progress, and some preclinical trials showed its promising therapeutic efficiency because of low immunogenicity and lower toxicity when compared with the cellular therapy [160–163].

## 7. Conclusion

In conclusion, mammalian cells communicate with distant cells by secreting metabolic products, including EVs. To date, the findings from many studies provide evidence that firmly establishes EVs as mediators of cell-cell communication. Retrieval of EVs from biological fluids for use as biomarkers in disease diagnosis is an innovative and appealing idea. Stressed stem cells and other differentiated cells release EVs containing promising protective messengers (e.g., proteins, mRNA, miRNA, and metabolites) that can be delivered to other cells. Extracellular vesicles are secreted in large amounts in biological fluids, particularly in cancer, and could provide a minimally invasive method for detecting disease or assessing disease progression. Further studies are required to bring this potentially valuable tool into use in clinical practice. Finally, stress-derived EVs may be used for therapeutic purposes and as protective means against certain kinds of physical and chemical stressors, including the air pollution. Molecular investigations into cellular stress-derived EVs could provide a paradigm for manufacturing artificial exosome-like nanoparticles for drug delivery and as therapeutics.

## Figures and Tables

**Table 1:** Types and characteristics of different EVs.

Characteristic	Exosomes	Microvesicles	Apoptotic bodies
Size (nm)	30-180	100-1000	500-2000
Origin	MVB exocytosis	Cell membrane budding and fission	Plasma membrane, endoplasmic reticulum
Morphology	Cup or round shaped	Various shapes	Various shapes
Surface markers	Annexins, tetraspanins, and heat-shock proteins	CD40, cholesterol, sphingomyelin, and ceramide	Annexin V positivity, TSP, and C3b
Contents	Proteins, nucleic acids, lipid	Proteins, nucleic acids, lipid	Nuclear fractions, DNA, cell organelles

**Table 2:** EV contents help target cell against stressors.

Stressor	Cell of origin	EV cargo contents	Target cell	Reference
Physical	Gamma ray-irradiated lymphocytes	PI3K/AKT	Lymphocytes	[113]
	X-ray-irradiated human neuroblastoma SY5Y	AKT pathway	Nonirradiated SH-SY5Y	[164]
	Heat-shocked MCF-7 & K562	Unknown	Nonheat-shocked MCF-7 & K562	[1]
	Heat-shocked bovine granulosa	miRNAs	Nonheat-shocked bovine granulosa	[121]
	Heat-shocked bone marrow mesenchymal stem cells	HSP90 and HSP70	Granulosa cells	[155]
	Heat-shocked Sca-1(+) stem cells	HSF1, HSP70, and miR-34a	Cardiomyocytes	[154]
	Heat-treated gastric cancer	Hsp70 and Hsp60	Dendritic cells & cytotoxic T lymphocytes	[127]
	Heat-shocked colon adenocarcinoma	HSP70	Dendritic cells and regulatory T lymphocytes	[128]
	Lung carcinoma and melanoma	CCL2, CCL3, CCL4, CCL5, and CCL20, HSPs, and tumor antigens	Dendritic cells and cytotoxic T lymphocytes	[132]

Chemical	Oxidative bovine granulosa	Nrf2 (mRNA and protein) and antioxidant molecules (CAT, PRDX1, and TXN1)	Normal granulosa cells	[136]

Physiochemical	Heat stress+doxorubicin treated MCF-7 breast cancer	Caspase 3 and caspase 8	MCF-7 & in vivo (inhibited the growth of implanted breast tumors in mice)	[107]

**Table 3:** HSP extracellular vesicles in predictive and prognostic biomarker studies.

HSPs	Disease type	Sample type	Findings	Reference
HSP70	Carcinoma cells	Cell lines	Stimulate migration and cytolytic activity of natural killer cells	[51]
	Breast cancer	Plasma samples	Higher in breast and lung cancers, compared with control groups	[118]
	Lung cancer	Plasma samples	Increase in metastatic lung cancer	[118]
	Human prostate carcinoma	Cell lines	Recognition of the tumor cells by the immune system	[165]
	Breast, lung, and ovarian cancer	Urine samples	HSP70 exosomes were higher in cancer patients than in healthy donors	[166]

HSP90	Oral squamous cell carcinoma	Cell line	Lymph-node-metastatic	[26]

HSP70 and HSP90	Melanoma	Plasma from different disease stages	Disease progression and metastasis	[93]

HSP72	Thymoma, mammary carcinoma, and colon carcinoma	Mouse tumor cell lines	Stat3 activation (tumor survival mechanism and immunosuppressive function)	[87]

## Abbreviations

BE: Bystander effect
CAT: Catalase
CD: Cluster of differentiation
COPD: Chronic obstructive pulmonary disease
CS: Cigarette smoke
CTL: Cytotoxic T lymphocyte
DC: Dendritic cell
EMT: Epithelial-to-mesenchymal transition
EVs: Extracellular vesicles
HSC: Heat-shock cognate
HSP: Heat-shock protein
IL-6: Inteleukin-6
MAPK: Mitogen-activated protein kinase
MCF-7: Michigan Cancer Foundation-7 cell line
MHC: Major histocompatibility complex
miRNA: MicroRNA
mRNA: Messenger RNA
MUC: Mucin
MVBs: Multivesicular bodies
NRF2: Nuclear factor erythroid 2-related factor 2
PM: Particulate matter
PRDX1: Peroxiredoxin 1
Rab: Ras-associated binding protein
ROS: Reactive oxygen species
TXN1: Thioredoxin 1.

## References

[1] Bewicke-Copley F., Mulcahy L. A., Jacobs L. A. (2017). Extracellular vesicles released following heat stress induce bystander effect in unstressed populations. *Journal of Extracellular Vesicles*.

[2] Chakraborty A., Held K. D., Prise K. M., Liber H. L., Redmond R. W. (2009). Bystander effects induced by diffusing mediators after photodynamic stress. *Radiation Research*.

[3] Dabrowska A., Goś M., Janik P. (2005). "Bystander effect" induced by photodynamically or heat-injured ovarian carcinoma cells (OVP10) in vitro. *Medical Science Monitor*.

[4] Purschke M., Laubach H. J., Rox Anderson R., Manstein D. (2010). Thermal injury causes DNA damage and lethality in unheated surrounding cells: active thermal bystander effect. *The Journal of Investigative Dermatology*.

[5] Martin O. A., Nowsheen S., Siva S., Aziz K., Hatzi V. I., Georgakilas A. G., Preedy V. (2014). Inflammation and Oxidative DNA Damage: A Dangerous Synergistic Pathway to Cancer. *Cancer*.

[6] Floeth F. W., Shand N., Bojar H. (2001). Local inflammation and devascularization -- *in vivo* mechanisms of the "bystander effect" in VPC-mediated HSV-Tk/GCV gene therapy for human malignant glioma. *Cancer Gene Therapy*.

[7] Najafi M., Fardid R., Hadadi G., Fardid M. (2014). The mechanisms of radiation-induced bystander effect. *Journal of Biomedical Physics and Engineering*.

[8] Di X., Bright A. T., Bellott R. (2008). A chemotherapy-associated senescence bystander effect in breast cancer cells. *Cancer Biology & Therapy*.

[9] Merle P., Morvan D., Caillaud D., Demidem A. (2008). Chemotherapy-induced bystander effect in response to several chloroethylnitrosoureas: an origin independent of DNA damage?. *Anticancer Research*.

[10] Garcia-Rodríguez L., Abate-Daga D., Rojas A., González J. R., Fillat C. (2011). E-cadherin contributes to the bystander effect of TK/GCV suicide therapy and enhances its antitumoral activity in pancreatic cancer models. *Gene Therapy*.

[11] Mesnil M., Yamasaki H. (2000). Bystander effect in herpes simplex virus-thymidine kinase/ganciclovir cancer gene therapy: role of gap-junctional intercellular communication. *Cancer Research*.

[12] Das J. K., Xiong X., Ren X., Yang J. M., Song J. (2019). Heat shock proteins in cancer immunotherapy. *Journal of Oncology*.

[13] Gao W., Li L., Wang Y. (2015). Bronchial epithelial cells: the key effector cells in the pathogenesis of chronic obstructive pulmonary disease?. *Respirology*.

[14] Randell S. H. (2006). Airway epithelial stem cells and the pathophysiology of chronic obstructive pulmonary disease. *Proceedings of the American Thoracic Society*.

[15] Schraufnagel D. E., Blasi F., Drummond M. B. (2014). Electronic cigarettes. A position statement of the forum of international respiratory societies. *American Journal of Respiratory and Critical Care Medicine*.

[16] Yan R., Chen X. L., Xu Y. M., Lau A. T. Y. (2021). Epimutational effects of electronic cigarettes. *Environmental Science and Pollution Research International*.

[17] Huang S. J., Xu Y. M., Lau A. T. Y. (2018). Electronic cigarette: a recent update of its toxic effects on humans. *Journal of Cellular Physiology*.

[18] Savukinas U. B., Enes S. R., Sjöland A. A., Westergren-Thorsson G. (2016). Concise review: the bystander effect: mesenchymal stem cell-mediated lung repair. *Stem Cells*.

[19] Laskey R. A., Honda B. M., Mills A. D., Finch J. T. (1978). Nucleosomes are assembled by an acidic protein which binds histones and transfers them to DNA. *Nature*.

[20] Jolly C., Morimoto R. I. (2000). Role of the heat shock response and molecular chaperones in oncogenesis and cell death. *Journal of the National Cancer Institute*.

[21] Lang B. J., Guerrero-Giménez, Prince, Ackerman, Bonorino, Calderwood (2019). Heat shock proteins are essential components in transformation and tumor progression: cancer cell intrinsic pathways and beyond. *International Journal of Molecular Sciences*.

[22] Scieglinska D., Krawczyk Z., Sojka D. R., Gogler-Pigłowska A. (2019). Heat shock proteins in the physiology and pathophysiology of epidermal keratinocytes. *Cell Stress & Chaperones*.

[23] Seigneuric R., Mjahed H., Gobbo J. (2011). Heat shock proteins as danger signals for cancer detection. *Frontiers in Oncology*.

[24] Calderwood S. K., Gong J., Murshid A. (2016). Extracellular HSPs: the complicated roles of extracellular HSPs in immunity. *Frontiers in Immunology*.

[25] Clayton A., Turkes A., Navabi H., Mason M. D., Tabi Z. (2005). Induction of heat shock proteins in B-cell exosomes. *Journal of Cell Science*.

[26] Ono K., Eguchi T., Sogawa C. (2018). HSP-enriched properties of extracellular vesicles involve survival of metastatic oral cancer cells. *Journal of Cellular Biochemistry*.

[27] Eguchi T., Sogawa C., Okusha Y. (2018). Organoids with cancer stem cell-like properties secrete exosomes and HSP90 in a 3D nanoenvironment. *PLoS One*.

[28] Alzahrani F. A., Saadeldin I. M. (2021). *Role of Exosomes in Biological Communication Systems*.

[29] Théry C., Witwer K. W., Aikawa E. (2018). Minimal information for studies of extracellular vesicles 2018 (MISEV2018): a position statement of the International Society for Extracellular Vesicles and update of the MISEV2014 guidelines. *Journal of Extracellular Vesicles*.

[30] Willms E., Cabañas C., Mäger I., Wood M. J. A., Vader P. (2018). Extracellular vesicle heterogeneity: subpopulations, isolation techniques, and diverse functions in cancer progression. *Frontiers in Immunology*.

[31] Crescitelli R., Lässer C., Szabó T. G. (2013). Distinct RNA profiles in subpopulations of extracellular vesicles: apoptotic bodies, microvesicles and exosomes. *Journal of Extracellular Vesicles*.

[32] van Niel G., D'Angelo G., Raposo G. (2018). Shedding light on the cell biology of extracellular vesicles. *Nature Reviews Molecular Cell Biology*.

[33] Kowal J., Tkach M., Thery C. (2014). Biogenesis and secretion of exosomes. *Current Opinion in Cell Biology*.

[34] Deregibus M. C., Cantaluppi V., Calogero R. (2007). Endothelial progenitor cell–derived microvesicles activate an angiogenic program in endothelial cells by a horizontal transfer of mRNA. *Blood*.

[35] Colombo M., Raposo G., Théry C. (2014). Biogenesis, secretion, and intercellular interactions of exosomes and other extracellular vesicles. *Annual Review of Cell and Developmental Biology*.

[36] Lo Cicero A., Stahl P. D., Raposo G. (2015). Extracellular vesicles shuffling intercellular messages: for good or for bad. *Current Opinion in Cell Biology*.

[37] Raposo G., Stoorvogel W. (2013). Extracellular vesicles: exosomes, microvesicles, and friends. *Journal of Cell Biology*.

[38] Beit-Yannai E., Tabak S., Stamer W. D. (2018). Physical exosome:exosome interactions. *Journal of Cellular and Molecular Medicine*.

[39] Eitan E., Suire C., Zhang S., Mattson M. P. (2016). Impact of lysosome status on extracellular vesicle content and release. *Ageing Research Reviews*.

[40] Sidhom K., Obi P. O., Saleem A. (2020). A review of exosomal isolation methods: is size exclusion chromatography the best option?. *International Journal of Molecular Sciences*.

[41] Zhang Y., Bi J., Huang J., Tang Y., du S., Li P. (2020). Exosome: a review of its classification, isolation techniques, storage, diagnostic and targeted therapy applications. *International Journal of Nanomedicine*.

[42] Yang E., Wang X., Gong Z., Yu M., Wu H., Zhang D. (2020). Exosome-mediated metabolic reprogramming: the emerging role in tumor microenvironment remodeling and its influence on cancer progression. *Signal Transduction and Targeted Therapy*.

[43] Chatterjee S., Burns T. F. (2017). Targeting heat shock proteins in cancer: a promising therapeutic approach. *International Journal of Molecular Sciences*.

[44] Schmitt E., Gehrmann M., Brunet M., Multhoff G., Garrido C. (2007). Intracellular and extracellular functions of heat shock proteins: repercussions in cancer therapy. *Journal of Leukocyte Biology*.

[45] Jee H. (2016). Size dependent classification of heat shock proteins: a mini-review. *Journal of Exercise Rehabilitation*.

[46] Zininga T., Ramatsui L., Shonhai A. (2018). Heat shock proteins as immunomodulants. *Molecules*.

[47] Lanneau D., Brunet M., Frisan E., Solary E., Fontenay M., Garrido C. (2008). Heat shock proteins: essential proteins for apoptosis regulation. *Journal of Cellular and Molecular Medicine*.

[48] Voos W. (2013). Chaperone-protease networks in mitochondrial protein homeostasis. *Biochimica et Biophysica Acta (BBA) - Molecular Cell Research*.

[49] Basu S., Binder R. J., Suto R., Anderson K. M., Srivastava P. K. (2000). Necrotic but not apoptotic cell death releases heat shock proteins, which deliver a partial maturation signal to dendritic cells and activate the NF-kappa B pathway. *International Immunology*.

[50] Wyciszkiewicz A., Kalinowska-Łyszczarz A., Nowakowski B., Kaźmierczak K., Osztynowicz K., Michalak S. (2019). Expression of small heat shock proteins in exosomes from patients with gynecologic cancers. *Scientific Reports*.

[51] Gastpar R., Gehrmann M., Bausero M. A. (2005). Heat shock protein 70 surface-positive tumor exosomes stimulate migratory and cytolytic activity of natural killer cells. *Cancer Research*.

[52] Xu Q. (2002). Role of heat shock proteins in atherosclerosis. *Arteriosclerosis, Thrombosis, and Vascular Biology*.

[53] Richter K., Haslbeck M., Buchner J. (2010). The heat shock response: life on the verge of death. *Molecular Cell*.

[54] Schmidt E. E., Pelz O., Buhlmann S., Kerr G., Horn T., Boutros M. (2013). GenomeRNAi: a database for cell-based and in vivo RNAi phenotypes, 2013 update. *Nucleic Acids Research*.

[55] Daugaard M., Rohde M., Jaattela M. (2007). The heat shock protein 70 family: highly homologous proteins with overlapping and distinct functions. *FEBS Letters*.

[56] Taha E. A., Ono K., Eguchi T. (2019). Roles of extracellular HSPs as biomarkers in immune surveillance and immune evasion. *International Journal of Molecular Sciences*.

[57] Dubey A., Prajapati K. S., Swamy M., Pachauri V. (2015). Heat shock proteins: a therapeutic target worth to consider. *Vet World*.

[58] Graner M. W., Cumming R. I., Bigner D. D. (2007). The heat shock response and chaperones/heat shock proteins in brain tumors: surface expression, release, and possible immune consequences. *The Journal of Neuroscience*.

[59] Joly A. L., Wettstein G., Mignot G., Ghiringhelli F., Garrido C. (2010). Dual role of heat shock proteins as regulators of apoptosis and innate immunity. *Journal of Innate Immunity*.

[60] Dudeja V., Mujumdar N., Phillips P. (2009). Heat shock protein 70 inhibits apoptosis in cancer cells through simultaneous and independent mechanisms. *Gastroenterology*.

[61] Beere H. M., Wolf B. B., Cain K. (2000). Heat-shock protein 70 inhibits apoptosis by preventing recruitment of procaspase-9 to the Apaf-1 apoptosome. *Nature Cell Biology*.

[62] Gupta A., Sharma D., Gupta H. (2021). Heat precondition is a potential strategy to combat hepatic injury triggered by severe heat stress. *Life Sciences*.

[63] Kalmar B., Greensmith L. (2017). Cellular chaperones as therapeutic targets in ALS to restore protein homeostasis and improve cellular function. *Frontiers in Molecular Neuroscience*.

[64] Srivastava P. (2002). Interaction of heat shock proteins with peptides and antigen presenting cells: chaperoning of the innate and adaptive immune responses. *Annual Review of Immunology*.

[65] De Maio A. (2011). Extracellular heat shock proteins, cellular export vesicles, and the Stress Observation System: a form of communication during injury, infection, and cell damage. It is never known how far a controversial finding will go! Dedicated to Ferruccio Ritossa. *Cell Stress & Chaperones*.

[66] Vega V. L., Rodríguez-Silva M., Frey T. (2008). Hsp70 translocates into the plasma membrane after stress and is released into the extracellular environment in a membrane-associated form that activates macrophages. *Journal of Immunology*.

[67] Gruden G., Carucci P., Lolli V. (2013). Serum heat shock protein 27 levels in patients with hepatocellular carcinoma. *Cell Stress & Chaperones*.

[68] Caruso Bavisotto C., Cipolla C., Graceffa G. (2019). Immunomorphological pattern of molecular chaperones in normal and pathological thyroid tissues and circulating exosomes: potential use in clinics. *International Journal of Molecular Sciences*.

[69] Liao W.-C., Wu M. S., Wang H. P., Tien Y. W., Lin J. T. (2009). Serum heat shock protein 27 is increased in chronic pancreatitis and pancreatic carcinoma. *Pancreas*.

[70] Cui X., Huang M., Wang S. (2021). Circulating exosomes from patients with Graves' disease induce an inflammatory immune response. *Endocrinology*.

[71] van Noort J. M., Bsibsi M., Nacken P., Gerritsen W. H., Amor S. (2012). The link between small heat shock proteins and the immune system. *The International Journal of Biochemistry & Cell Biology*.

[72] Batulan Z., Venu V. K. P., Li Y. (2016). Extracellular release and signaling by heat shock protein 27: role in modifying vascular inflammation. *Frontiers in Immunology*.

[73] Villarroya-Beltri C., Gutiérrez-Vázquez C., Sánchez-Cabo F. (2013). Sumoylated hnRNPA2B1 controls the sorting of miRNAs into exosomes through binding to specific motifs. *Nature Communications*.

[74] Chen Y.-R., Sekine K., Nakamura K., Yanai H., Tanaka M., Miyajima A. (2009). Y-box binding protein-1 down-regulates expression of carbamoyl phosphate synthetase-I by suppressing CCAAT enhancer-binding protein-alpha function in mice. *Gastroenterology*.

[75] Arroyo J. D., Chevillet J. R., Kroh E. M. (2011). Argonaute2 complexes carry a population of circulating microRNAs independent of vesicles in human plasma. *Proceedings of the National Academy of Sciences of the United States of America*.

[76] Moreno-Gonzalo O., Fernandez-Delgado I., Sanchez-Madrid F. (2018). Post-translational add-ons mark the path in exosomal protein sorting. *Cellular and Molecular Life Sciences*.

[77] Zhang Y., Liu Y., Liu H., Tang W. H. (2019). Exosomes: biogenesis, biologic function and clinical potential. *Cell & Bioscience*.

[78] Zhang W., Ou X., Wu X. (2019). Proteomics profiling of plasma exosomes in epithelial ovarian cancer: a potential role in the coagulation cascade, diagnosis and prognosis. *International Journal of Oncology*.

[79] Alharbi M., Lai A., Guanzon D. (2019). Ovarian cancer-derived exosomes promote tumour metastasis in vivo: an effect modulated by the invasiveness capacity of their originating cells. *Clinical Science (London, England)*.

[80] Gangoda L., Liem M., Ang C. S. (2017). Proteomic profiling of exosomes secreted by breast cancer cells with varying metastatic potential. *Proteomics*.

[81] Anderson J. D., Johansson H. J., Graham C. S. (2016). Comprehensive proteomic analysis of mesenchymal stem cell exosomes reveals modulation of angiogenesis via nuclear factor-KappaB signaling. *Stem Cells*.

[82] Gangoda L., Boukouris S., Liem M., Kalra H., Mathivanan S. (2015). Extracellular vesicles including exosomes are mediators of signal transduction: are they protective or pathogenic?. *Proteomics*.

[83] Abramowicz A., Widłak P., Pietrowska M. (2019). Different types of cellular stress affect the proteome composition of small extracellular vesicles: a mini review. *Proteomes*.

[84] de Jong O. G., Verhaar M. C., Chen Y. (2012). Cellular stress conditions are reflected in the protein and RNA content of endothelial cell-derived exosomes. *Journal of Extracellular Vesicles*.

[85] Gehrmann M., Specht H. M., Bayer C. (2014). Hsp70--a biomarker for tumor detection and monitoring of outcome of radiation therapy in patients with squamous cell carcinoma of the head and neck. *Radiation Oncology*.

[86] Kahroba H., Hejazi M. S., Samadi N. (2019). Exosomes: from carcinogenesis and metastasis to diagnosis and treatment of gastric cancer. *Cellular and Molecular Life Sciences*.

[87] Chalmin F., Ladoire S., Mignot G. (2010). Membrane-associated Hsp72 from tumor-derived exosomes mediates STAT3-dependent immunosuppressive function of mouse and human myeloid-derived suppressor cells. *The Journal of Clinical Investigation*.

[88] Sheller S., Papaconstantinou J., Urrabaz-Garza R. (2016). Amnion-epithelial-cell-derived exosomes demonstrate physiologic state of cell under oxidative stress. *PLoS One*.

[89] Biasutto L., Chiechi A., Couch R., Liotta L. A., Espina V. (2013). Retinal pigment epithelium (RPE) exosomes contain signaling phosphoproteins affected by oxidative stress. *Experimental Cell Research*.

[90] Javeed N., Mukhopadhyay D. (2017). Exosomes and their role in the micro-/macro-environment: a comprehensive review. *Journal of Biomedical Research*.

[91] Chen W., Wang J., Shao C. (2006). Efficient induction of antitumor T cell immunity by exosomes derived from heat-shocked lymphoma cells. *European Journal of Immunology*.

[92] Cordonnier M., Chanteloup G., Isambert N. (2017). Exosomes in cancer theranostic: diamonds in the rough. *Cell Adhesion & Migration*.

[93] Peinado H., Alečković M., Lavotshkin S. (2012). Melanoma exosomes educate bone marrow progenitor cells toward a pro- metastatic phenotype through MET. *Nature Medicine*.

[94] McCready J., Sims J. D., Chan D., Jay D. G. (2010). Secretion of extracellular hsp90*α* via exosomes increases cancer cell motility: a role for plasminogen activation. *BMC Cancer*.

[95] Ham S., Lima L. G., Chai E. P. Z. (2018). Breast cancer-derived exosomes alter macrophage polarization via gp130/STAT3 signaling. *Frontiers in Immunology*.

[96] Kanlikilicer P., Bayraktar R., Denizli M. (2018). Exosomal miRNA confers chemo resistance via targeting Cav1/p-gp/M2-type macrophage axis in ovarian cancer. *eBioMedicine*.

[97] Wang X., Luo G., Zhang K. (2018). Hypoxic tumor-derived exosomal miR-301a mediates M2 macrophage polarization via PTEN/PI3K*γ* to promote pancreatic cancer metastasis. *Cancer Research*.

[98] Ariyoshi K., Miura T., Kasai K., Fujishima Y., Nakata A., Yoshida M. (2019). Radiation-induced bystander effect is mediated by mitochondrial DNA in exosome-like vesicles. *Scientific Reports*.

[99] Andre F., Schartz N. E. C., Movassagh M. (2002). Malignant effusions and immunogenic tumour-derived exosomes. *The Lancet*.

[100] Wolfers J., Lozier A., Raposo G. (2001). Tumor-derived exosomes are a source of shared tumor rejection antigens for CTL cross-priming. *Nature Medicine*.

[101] Alharbi M., Zuñiga F., Elfeky O. (2018). The potential role of miRNAs and exosomes in chemotherapy in ovarian cancer. *Endocrine-Related Cancer*.

[102] Crow J., Atay S., Banskota S., Artale B., Schmitt S., Godwin A. K. (2017). Exosomes as mediators of platinum resistance in ovarian cancer. *Oncotarget*.

[103] Chanteloup G., Cordonnier M., Isambert N. (2020). Monitoring HSP70 exosomes in cancer patients' follow up: a clinical prospective pilot study. *Journal of Extracellular Vesicles*.

[104] Alharbi M., Sharma S., Guanzon D. (2020). miRNa signature in small extracellular vesicles and their association with platinum resistance and cancer recurrence in ovarian cancer. *Nanomedicine: Nanotechnology, Biology and Medicine*.

[105] Keklikoglou I., Cianciaruso C., Güç E. (2019). Chemotherapy elicits pro-metastatic extracellular vesicles in breast cancer models. *Nature Cell Biology*.

[106] Patel G. K., Khan M. A., Bhardwaj A. (2017). Exosomes confer chemoresistance to pancreatic cancer cells by promoting ROS detoxification and miR-155-mediated suppression of key gemcitabine- metabolising enzyme, DCK. *British Journal of Cancer*.

[107] Yang Y., Chen Y., Zhang F., Zhao Q., Zhong H. (2015). Increased anti-tumour activity by exosomes derived from doxorubicin-treated tumour cells via heat stress. *International Journal of Hyperthermia*.

[108] O’Neill C., Gilligan K., Dwyer R. (2019). Role of extracellular vesicles (EVs) in cell stress response and resistance to cancer therapy. *Cancers*.

[109] Szatmári T., Hargitai R., Sáfrány G., Lumniczky K. (2019). Extracellular vesicles in modifying the effects of ionizing radiation. *International Journal of Molecular Sciences*.

[110] Preston R. J. (2005). Bystander effects, genomic instability, adaptive response, and cancer risk assessment for radiation and chemical exposures. *Toxicology and Applied Pharmacology*.

[111] Szatmári T., Persa E., Kis E. (2019). Extracellular vesicles mediate low dose ionizing radiation-induced immune and inflammatory responses in the blood. *International Journal of Radiation Biology*.

[112] Jabbari N., Nawaz M., Rezaie J. (2019). Ionizing radiation increases the activity of exosomal secretory pathway in MCF-7 human breast cancer cells: a possible way to communicate resistance against radiotherapy. *International Journal of Molecular Sciences*.

[113] Shankar B., Pandey R., Sainis K. (2006). Radiation-induced bystander effects and adaptive response in murine lymphocytes. *International Journal of Radiation Biology*.

[114] Sarin S. K., Chari S., Sundaram K. R., Ahuja R. K., Anand B. S., Broor S. L. (1988). Young v adult cirrhotics: a prospective, comparative analysis of the clinical profile, natural course and survival. *Gut*.

[115] Dai J., Su Y., Zhong S. (2020). Exosomes: key players in cancer and potential therapeutic strategy. *Signal Transduction and Targeted Therapy*.

[116] Tang X., Chang C., Guo J. (2019). Tumour-secreted Hsp90*α* on external surface of exosomes mediates tumour - stromal cell communication via autocrine and paracrine mechanisms. *Scientific Reports*.

[117] Eguchi T., Sogawa C., Ono K. (2020). Cell stress induced stressome release including damaged membrane vesicles and extracellular HSP90 by prostate cancer cells. *Cells*.

[118] Chanteloup G., Cordonnier M., Isambert N. (2020). Membrane-bound exosomal HSP70 as a biomarker for detection and monitoring of malignant solid tumours: a pilot study. *Pilot Feasibility Study*.

[119] Heads R. J., Yellon D. M., Latchman D. S. (1995). Differential cytoprotection against heat stress or hypoxia following expression of specific stress protein genes in myogenic cells. *Journal of Molecular and Cellular Cardiology*.

[120] Mehla K., Magotra A., Choudhary J. (2014). Genome-wide analysis of the heat stress response in Zebu (Sahiwal) cattle. *Gene*.

[121] Gebremedhn S., Gad A., Aglan H. S. (2020). Extracellular vesicles shuttle protective messages against heat stress in bovine granulosa cells. *Scientific Reports*.

[122] Gebremedhn S., Ali A., Gad A., Prochazka R., Tesfaye D. (2020). Extracellular vesicles as mediators of environmental and metabolic stress coping mechanisms during mammalian follicular development. *Frontiers in Veterinary Science*.

[123] Xu D., Dong P., Xiong Y. (2020). MicroRNA-361-mediated inhibition of HSP90 expression and EMT in cervical cancer is counteracted by oncogenic lncRNA NEAT1. *Cell*.

[124] MacKenzie T. N., Mujumdar N., Banerjee S. (2013). Triptolide induces the expression of miR-142-3p: a negative regulator of heat shock protein 70 and pancreatic cancer cell proliferation. *Molecular Cancer Therapeutics*.

[125] Choghaei E., Khamisipour G., Falahati M. (2016). Knockdown of microRNA-29a changes the expression of heat shock proteins in breast carcinoma MCF-7 cells. *Oncology Research*.

[126] Chaput N. (2006). Dendritic cell derived-exosomes: biology and clinical implementations. *Journal of Leukocyte Biology*.

[127] Zhong H., Yang Y., Ma S. (2011). Induction of a tumour-specific CTL response by exosomes isolated from heat-treated malignant ascites of gastric cancer patients. *International Journal of Hyperthermia*.

[128] Guo D., Chen Y., Wang S. (2018). Exosomes from heat-stressed tumour cells inhibit tumour growth by converting regulatory T cells to Th17 cells via IL-6. *Immunology*.

[129] Hoter A., Alsantely A. O., Alsharaeh E., Kulik G., Saadeldin I. M. (2020). Combined Thermotherapy and Heat Shock Protein Modulation for Tumor Treatment. *Heat Shock Proteins in Human Diseases*.

[130] Toraya-Brown S., Fiering S. (2014). Local tumour hyperthermia as immunotherapy for metastatic cancer. *International Journal of Hyperthermia*.

[131] Skitzki J. J., Repasky E. A., Evans S. S. (2009). Hyperthermia as an immunotherapy strategy for cancer. *Current Opinion in Investigational Drugs*.

[132] Chen T., Guo J., Yang M., Zhu X., Cao X. (2011). Chemokine-containing exosomes are released from heat-stressed tumor cells via lipid raft-dependent pathway and act as efficient tumor vaccine. *The Journal of Immunology*.

[133] Eldh M., Ekström K., Valadi H. (2010). Exosomes communicate protective messages during oxidative stress; possible role of exosomal shuttle RNA. *PLoS One*.

[134] Nicholson C., Shah N., Ishii M. (2020). Mechanisms of extracellular vesicle uptake in stressed retinal pigment epithelial cell monolayers. *Biochimica et Biophysica Acta - Molecular Basis of Disease*.

[135] Shah N., Ishii M., Brandon C. (2018). Extracellular vesicle-mediated long-range communication in stressed retinal pigment epithelial cell monolayers. *Biochimica et Biophysica Acta - Molecular Basis of Disease*.

[136] Saeed-Zidane M., Linden L., Salilew-Wondim D. (2017). Cellular and exosome mediated molecular defense mechanism in bovine granulosa cells exposed to oxidative stress. *PLoS One*.

[137] Kesimer M., Scull M., Brighton B. (2009). Characterization of exosome-like vesicles released from human tracheobronchial ciliated epithelium: a possible role in innate defense. *The FASEB Journal*.

[138] Gupta R., Radicioni G., Abdelwahab S. (2019). Intercellular communication between airway epithelial cells is mediated by exosome-like vesicles. *American Journal of Respiratory Cell and Molecular Biology*.

[139] Comfort N., Smith C., Chillrud S., Yang Q., Baccarelli A., Jack D. (2019). Extracellular vesicles in saliva as biomarkers of exposure and effect. *Environmental Epidemiology*.

[140] Bourdonnay E., Zasłona Z., Penke L. R. K. (2015). Transcellular delivery of vesicular SOCS proteins from macrophages to epithelial cells blunts inflammatory signaling. *The Journal of Experimental Medicine*.

[141] Xu H., Ling M., Xue J. (2018). Exosomal microRNA-21 derived from bronchial epithelial cells is involved in aberrant epithelium-fibroblast cross-talk in COPD induced by cigarette smoking. *Theranostics*.

[142] He S., Chen D., Hu M. (2019). Bronchial epithelial cell extracellular vesicles ameliorate epithelial- mesenchymal transition in COPD pathogenesis by alleviating M2 macrophage polarization. *Nanomedicine: Nanotechnology, Biology and Medicine*.

[143] Fujita Y., Araya J., Ito S. (2015). Suppression of autophagy by extracellular vesicles promotes myofibroblast differentiation in COPD pathogenesis. *Journal of Extracellular Vesicles*.

[144] Phinney D. G., di Giuseppe M., Njah J. (2015). Mesenchymal stem cells use extracellular vesicles to outsource mitophagy and shuttle microRNAs. *Nature Communications*.

[145] Li L., Jin S., Zhang Y. (2015). Ischemic preconditioning potentiates the protective effect of mesenchymal stem cells on endotoxin-induced acute lung injury in mice through secretion of exosome. *International Journal of Clinical and Experimental Medicine*.

[146] Levänen B., Bhakta N. R., Torregrosa Paredes P. (2013). Altered microRNA profiles in bronchoalveolar lavage fluid exosomes in asthmatic patients. *The Journal of Allergy and Clinical Immunology*.

[147] Huang F., Jia H., Zou Y., Yao Y., Deng Z. (2020). Exosomes: an important messenger in the asthma inflammatory microenvironment. *Journal of International Medical Research*.

[148] Pua H. H., Ansel K. M. (2015). MicroRNA regulation of allergic inflammation and asthma. *Current Opinion in Immunology*.

[149] Maes T., Cobos F. A., Schleich F. (2016). Asthma inflammatory phenotypes show differential microRNA expression in sputum. *The Journal of Allergy and Clinical Immunology*.

[150] Pua H. H., Happ H. C., Gray C. J. (2019). Increased hematopoietic extracellular RNAs and vesicles in the lung during allergic airway responses. *Cell Reports*.

[151] Bryniarski K., Ptak W., Jayakumar A. (2013). Antigen-specific, antibody-coated, exosome-like nanovesicles deliver suppressor T-cell microRNA-150 to effector T cells to inhibit contact sensitivity. *Journal of Allergy and Clinical Immunology*.

[152] Benedikter B. J., Wouters E. F. M., Savelkoul P. H. M., Rohde G. G. U., Stassen F. R. M. (2018). Extracellular vesicles released in response to respiratory exposures: implications for chronic disease. *Journal of Toxicology and Environmental Health, Part B*.

[153] Schioppo T., Hoxha M., Iodice S. Ab0141 the role of air pollution on extracellular vesicles as a potential pro-inflammatory stimulus in rheumatoid arthritis.

[154] Feng Y., Huang W., Meng W. (2014). Heat shock improves Sca-1+stem cell survival and directs ischemic cardiomyocytes toward a prosurvival phenotype via exosomal transfer: a critical role for HSF1/miR-34a/HSP70 pathway. *Stem Cells*.

[155] Wang Q., Li X., Wang Q., Xie J., Xie C., Fu X. (2019). Heat shock pretreatment improves mesenchymal stem cell viability by heat shock proteins and autophagy to prevent cisplatin-induced granulosa cell apoptosis. *Stem Cell Research & Therapy*.

[156] du Y. M., Zhuansun Y. X., Chen R., Lin L., Lin Y., Li J. G. (2018). Mesenchymal stem cell exosomes promote immunosuppression of regulatory T cells in asthma. *Experimental Cell Research*.

[157] Liu C., Su C. (2019). Design strategies and application progress of therapeutic exosomes. *Theranostics*.

[158] Almughem F. A., Alshehri A. A., Alomary M. N., Alzahrani F. A., Saadeldin I. M. (2021). Exosomes in Drug Delivery. *Role of Exosomes in Biological Communication Systems*.

[159] Elnaggar M. A., Joung Y. K. (2021). Exosomes and Supported Lipid Layers as Advanced Naturally Derived Drug Delivery Systems. *Role of Exosomes in Biological Communication Systems*.

[160] Zhu X., Badawi M., Pomeroy S. (2017). Comprehensive toxicity and immunogenicity studies reveal minimal effects in mice following sustained dosing of extracellular vesicles derived from HEK293T cells. *Journal of Extracellular Vesicles*.

[161] Popowski K., Lutz H., Hu S., George A., Dinh P. U., Cheng K. (2020). Exosome therapeutics for lung regenerative medicine. *Journal of Extracellular Vesicles*.

[162] Wang N., Wang Q., du T. (2021). The potential roles of exosomes in chronic obstructive pulmonary disease. *Frontiers in Medicine*.

[163] Saleh A. F., Lázaro-Ibáñez E., Forsgard M. A. M. (2019). Extracellular vesicles induce minimal hepatotoxicity and immunogenicity. *Nanoscale*.

[164] Tortolici F., Vumbaca S., Incocciati B. (2021). Ionizing radiation-induced extracellular vesicle release promotes AKT-associated survival response in SH-SY5Y neuroblastoma cells. *Cells*.

[165] Mambula S. S., Calderwood S. K. (2006). Heat shock protein 70 is secreted from tumor cells by a nonclassical pathway involving lysosomal endosomes. *Journal of Immunology*.

[166] Gobbo J., Marcion G., Cordonnier M. (2016). Restoring anticancer immune response by targeting tumor-derived exosomes with a HSP70 peptide aptamer. *Journal of the National Cancer Institute*.

